# Solitary Testicular Metastasis From Prostate Cancer Treated With Orchiectomy: A Rare Case of Isolated Recurrence After Salvage Radiotherapy

**DOI:** 10.1155/criu/7042029

**Published:** 2025-10-15

**Authors:** Moritz Gutjahr-Holland, Shreya Armstrong

**Affiliations:** Department of Radiation Oncology, North Coast Cancer Institute, Lismore Base Hospital, Lismore, New South Wales, Australia

## Abstract

Prostate cancer (PCa) is the most commonly diagnosed cancer in Australia with almost 25,000 cases being diagnosed each year. Treatment for PCa varies depending on stage, patient preferences and the general health of the patient. PCa most commonly spreads to lymph nodes and bones. We present a case of a 66-year-old male who presented with PSA elevation post salvage radiation and was diagnosed with oligometastatic PCa to the right testis. This patient subsequently underwent a right orchidectomy resulting in a rapid fall in PSA.

## 1. Introduction

In Australia, prostate cancer is the most commonly diagnosed cancer [1] and is the cause of death of approximately 3700 men every year [2]. Risk factors for prostate cancer include advancing age; a positive family history; certain inherited genetic variants such as BRCA1/2, CHEK2 and HOXB13 [3] and ethnicity. Prostate cancer can be localised or metastatic at diagnosis. Approximately 10% of patients have metastatic prostate cancer at diagnosis [4] with the most common metastases found in the bones, lymph nodes, liver and lungs. Rarely does prostate cancer metastasise to the testis [5]. Rarer still do testicular metastases from prostate cancer occur in an oligometastatic fashion. We present a case of an elderly male who was diagnosed with oligometastatic prostate cancer to the right testis after salvage radiotherapy and subsequently underwent a right orchidectomy. Informed consent was obtained from the patient for publication of this case report.

## 2. Case Presentation

A 66-year-old male with a history of treated prostate cancer was being followed up in the radiation oncology outpatients clinic. He had been first diagnosed with prostate cancer in 2020 after presenting with lower urinary tract symptoms (LUTSs). An elevated PSA to 5.6 *μ*g/L was identified, and the patient underwent a PSMA PET at the time, which demonstrated intense PSMA activity in the left peripheral zone of the prostate from base to mid gland, and a separate moderately PSMA avid focus in the right mid zone of the prostate. The patient proceeded to a laparoscopic prostatectomy in early 2021. Intraoperative findings demonstrated a large left posterior cancer. Histopathology demonstrated a Gleason 3 + 4 = 7 prostate cancer with a tumour volume of 9.05 cm^3^. There was lymphovascular invasion present, intraductal carcinoma present and established extra prostatic extension. The surgical margins were involved. There was carcinomatous involvement of the left and right neurovascular bundles and bilaterally extensive involvement of the seminal vesicles. Postoperatively, the PSA fell to 0.28 at 6 weeks but began climbing to 0.35 *μ*g/L (24/6/2021) at 3 months, giving an estimated PSA doubling time of approximately 5 months. A repeat PSMA PET was undertaken in July 2021, which demonstrated no abnormal PSMA uptake. After ongoing PSA rise to 0.5 *μ*g/L (14/10/2021) the patient commenced salvage radiation in late 2021 (64Gy in 32#) with 6 months of androgen deprivation therapy (ADT). PSA was suppressed on ADT with a nadir of 0.01 *μ*g/L.

**Figure 1:**
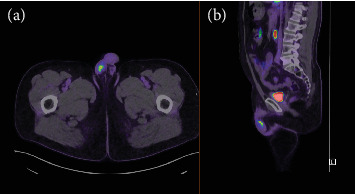
PSMA PET. Axial slice (a). Sagittal slice (b). Avid region correlates with right testis.

**Figure 2:**
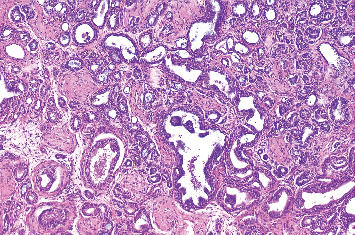
HE-stained section of the resected specimen. The pathological findings indicate metastatic prostate cancer.

Unfortunately, in early 2023, this patient's PSA began to rise and reached 0.77 *μ*g/L (19/1/2024). A further PSMA PET was performed which demonstrated a single hotspot in the right testis (Figure 1). Consistent with this, a clinical examination demonstrated a tender and swollen right testis. His case was discussed at a multidisciplinary team with a recommendation for a referral for consideration of orchidectomy. The patient underwent a right radical orchiectomy on 12/02/2024. The operation was completed without complication and the patient was discharged from hospital the day of surgery. Histopathology of the orchiectomy specimen demonstrated nodules of metastatic prostatic adenocarcinoma in the right testis (Figure 2) with lymphovascular invasion. The tumour was graded as Gleason 4 + 3 = 7. This was excised clear of margins. Postoperatively, PSA fell to 0.19 *μ*g/L on 26/02/2024 and further to 0.12 *μ*g/L on 11/4/2024, approximately 2.5 months after surgery. Subsequently, the PSA demonstrates an upwards trend to 0.29 *μ*g/L (31/03/25) and most recently to 0.38 *μ*g/L (08/07/25). The patient is well and continues on regular 3 monthly PSA surveillance with a plan to repeat PSMA PET imaging if the PSA reaches 0.5 *μ*g/L.

## 3. Discussion

It is well known that prostate cancer typically metastasises to bones, lymph nodes, liver and lungs [6]. However, metastatic disease to the testis is rare. The earliest published report of secondary metastasis to the testicle from another primary site was described by Semans in 1938 [7]. Large autopsy reviews have since confirmed its rarity; one study of 24,000 autopsies demonstrated only 15 cases of testicular metastases with an incidence of 0.06% [8]. The primary sources of testicular secondaries are the prostate, colon, kidney, stomach, pancreas and melanoma [9]. Reports of oligometastatic prostate cancer to the testis in the literature are limited to case reports, the first of which was published in 1984 [10]. Testicular metastases have been associated with a poor prognosis; a Taiwanese series of seven cases demonstrated survival around 12 months [11]. Possible pathways of spread of PCa to the testis have been speculated to include haematogenous spread, including arterial embolism, lymphatic spread or endocanalicular spread [12]. Patients with secondaries in the testis may present with a testicular mass, testicular swelling or testicular pain [13] but may also be asymptomatic [12]. However, it is important to keep in mind that in younger men, the main oncological differential for a testicular mass would be primary testicular cancer, as it is the most common tumour in the 15–34 year age group [14]. The most common testicular cancer is a germ cell tumour.

Although the precise route of spread in our patient cannot be confirmed, it is worth considering whether the prior laparoscopic prostatectomy might have played a role. Surgical manipulation, particularly when the neurovascular bundles and seminal vesicles are extensively involved by tumour, could theoretically facilitate the dissemination of malignant cells. Mechanisms could include tumour cell spillage into lymphatic, vascular or endocanalicular channels (vas deferens) during the dissection, or microscopic seeding along surgical pathways. However, such events remain highly speculative and there is currently no direct evidence linking laparoscopic radical prostatectomy to testicular metastases. In this context, it is the opinion of the authors that the most likely route of metastasis is via haematogenous spread.

Prostate cancer is highly survivable, with a survival rate of 95% at 5 years [2]. However, the presence of metastasis reduces this significantly. According to data from the American Cancer Society, metastatic prostate cancer carries a 34% 5-year survival. Despite this, metastatic prostate cancer has a number of treatment options. These include watchful waiting, radiation therapy, hormone therapy, chemotherapy and radioisotope therapy. As the next paragraph will discuss, treatments for metastatic prostate cancer have improved in recent years. Thus, men being diagnosed with prostate cancer currently could be expected to have a better survival than the aforementioned numbers would indicate.

Patients with metastatic cancer that is confined to a limited number of sites (oligometastasis), such as our patient, may have the unique opportunity of metastasis-directed therapy (MDT). The theory behind MDT is to eliminate the limited metastatic disease before the tumour cells can select for aggressive clonagens that have the ability and propensity to metastasise to further distant sites [15]. In selected patients, the MDT approach can potentially be curable. The optimal treatment approach for MDT is debated and includes ablative radiotherapy (SABR/SBRT), metastatectomy (as occurred here), radioligand therapy and observation. Evidence supporting MDT includes the phase II ORIOLE trial, which randomised 54 men to either SBRT or observation. Treated patients had significantly lower rates of disease progression compared with controls (61% vs. 19%) [16]. The benefit, however, relies heavily on accurate detection of all metastatic sites. The presence of even a *single* untreated metastasis was associated with worse median distant metastasis-free survival (6 months vs. 29 months) [16]. Further evidence for the durability of MDT is provided by a retrospective study by Milenkovic et al. of 211 patients with oligorecurrent PCa after radical prostatectomy, whereupon delivery of MDT, 23% of patients in this study remained free of a second recurrence at 5 years [17]

Fortunately, modern molecular imaging techniques such as PSMA-PET have significantly improved the detection of metastasis with high sensitivity and specificity [18]. This imaging technique was used in our patient to detect the solitary testicular metastasis. The main weakness of this technique is the sensitivity, with studies reporting 75%–85% sensitivity [18, 19].

Another benefit of MDT is that it may enable men to avoid or delay the use of ADT [16, 17, 20, 21]. ADT carries a broad side effect profile, particularly in younger men, and carries long-term adverse health effects including reduction in bone density, decrease in muscle mass and weight gain which may predispose to metabolic syndrome. Many patients would be interested in avoiding these side effects. The main limitation of the MDT approach would be the potential for acute toxicities from the local therapy. Fortunately, unilateral orchiectomy is generally well tolerated, and indeed, our patient was discharged home the day of surgery.

## 4. Conclusion

Metastatic prostate cancer to the testis is a rare diagnosis, especially so when it occurs in an isolated and oligometastatic fashion. Despite this, in oncological patients with testicular masses or pain, the possibility of testicular secondaries must be considered. The pathways for this form of metastatic spread remain uncertain. Our patient underwent a unilateral orchidectomy with rapid response in serum PSA. This is a pleasing result, and it is hoped that this will prevent progression of metastatic disease and may also allow our patient to delay the introduction of ADT. Our patient is undergoing ongoing PSA surveillance to assess if further treatments or investigations are required.

## Data Availability

The data that support the findings of this study are available on request from the corresponding author. The data are not publicly available due to privacy or ethical restrictions.
